# Molecular dynamics investigation of interfacial energy and mechanical behavior in braid-reinforced hollow fiber membranes

**DOI:** 10.1038/s41598-026-41106-0

**Published:** 2026-07-08

**Authors:** Mostafa Jafari, Ali Vatani, Toraj Mohammadi, Ahmadreza Andarz

**Affiliations:** 1 School of Chemical Engineering, College of Engineering, University of Tehran, Tehran, Iran; 2 Institute of Liquefied Natural Gas (I-LNG), School of Chemical Engineering, College of Engineering, University of Tehran, Tehran, Iran; 3 Center of Excellence for Membrane Research and Technology, Department of Chemical, Petroleum and Gas Engineering, Iran University of Science and Technology (IUST), Narmak, Tehran, Iran

**Keywords:** Numerical simulation, Interfacial properties, Composites, Reinforcements, Polymers, Chemistry, Engineering, Materials science

## Abstract

This study presents a molecular dynamics investigation of braid-reinforced hollow fiber membranes to elucidate the interfacial and mechanical behaviors of polymeric composites composed of cellulose acetate (CA) and polyacrylonitrile (PAN). The analysis focuses on three representative configurations, homogeneous (CA/CA-II), semi-heterogeneous (PAN|CA/CA-I), and heterogeneous hybrid (CA/PAN-III), to evaluate their interfacial energies, adhesion mechanisms, and tensile responses. The calculated interfacial energies of − 6.32077, − 5.69262, and − 4.71113 mJ/m^2^ for CA/CA-II, PAN|CA/CA-I, and CA/PAN-III, respectively, reveal that chemical homogeneity promotes stronger interfacial bonding, whereas polarity mismatches between functional groups (–OH, –OCOCH_3_, and –CN) weaken adhesion and increase diffusivity at the interface. Mechanical testing through MD tensile simulations further demonstrates that the CA/PAN-III composite exhibits pronounced stress fluctuations and higher local interfacial activity. At the same time, the CA/CA-II system maintains the highest cohesive stability and elastic modulus due to structural uniformity. The CA/PAN-III hybrid achieves an optimal balance between flexibility and strength, indicating its suitability for water treatment membranes that require both mechanical resilience and interfacial durability. These findings provide molecular-level insight into how polymer compatibility governs the performance of braid-reinforced hollow fiber membranes and offer valuable guidelines for designing next-generation high-strength composite membranes.

## Introduction

The global water crisis, intensified by rapid population growth, industrial expansion, and climate change, has created an urgent demand for efficient and sustainable water purification technologies^1–3^. Among available approaches, membrane-based separation processes have emerged as among the most energy-efficient and environmentally friendly solutions for wastewater treatment and desalination, owing to their high selectivity, compact design, and operational flexibility^4–6^. In this field, membrane bioreactors (MBRs) have attracted significant attention because they combine biological degradation with membrane filtration, providing superior effluent quality and a smaller footprint thanonventional treatment systems^4,7^.

Hollow fiber membranes are the most common configuration used in MBRs, primarily because of their large surface-area-to-volume ratio and ease of module integration^8^. However, maintaining both sufficient mechanical strength and high permeability remains a significant challenge, especially under harsh industrial conditions involving pressure fluctuations, fouling, and long-term chemical exposure^9,10^. To overcome these limitations, braid-reinforced hollow fiber membranes (BRHFMs) have been developed, in which a braided fabric serves as a mechanical skeleton that enhances structural durability and prevents fiber collapse^11^. The overall performance of BRHFMs depends strongly on the interfacial adhesion between the polymer coating and the braid reinforcement, as well as on the degree of compatibility between the two materials^12^.

Extensive experimental research has focused on improving the mechanical integrity and permeability of composite membranes^13,14^. However, experimental methods alone often fail to capture the detailed interfacial mechanisms that govern adhesion, molecular diffusion, and stress transfer at the polymer–braid interface. Furthermore, achieving an optimal balance between interfacial bonding strength and water permeability remains difficult. Strong interfacial bonding can reduce porosity and limit water flux, whereas weak bonding may cause delamination or mechanical failure during operation^13,15^. In recent years, molecular dynamics (MD) simulations have become a robust computational method for studying molecular-scale interactions, nanotechnology, and underlying physical phenomena across different scientific fields^16–24^. Many researchers have used MD to investigate polymer–nanofiller interactions, interfacial adhesion in fiber-reinforced composites, and the deformation behavior of polymeric membranes^18,19,25,26^. In the context of interfacial energy and the mechanical properties of polymer composites, Li et al. provided valuable data regarding hollow fiber membranes^11,27^. However, to the best of the authors’ knowledge, systematic MD studies focusing specifically on BRHFMs for CA and PAN, particularly those examining the effects of homogeneous and heterogeneous polymer combinations on interfacial energy and mechanical properties, are still lacking in the literature.

MD simulation is a well-established method for investigating the mechanical^28^, thermal^29^, and interphase/interface^30^ properties of materials across scales from bulk to nanoscale. Modeling nanocomposites and determining their characteristics are achievable because the velocity and position of ions and bonds, collectively known as classical physical properties, can be obtained using this method. A literature review of nanocomposite modeling^20–23^, using polymers as the matrix and carbon allotropes as reinforcement, reveals that the reinforcement is typically defined first, followed by the matrix. Subsequently, the system was heated and equilibrated for a sufficient period to reach thermodynamic equilibrium. Following this, uniaxial strain was applied to the system, primarily along the reinforcement axis, and the resulting stress was computed. Mechanical properties, such as Young’s modulus and fracture points, were then calculated from the stress-strain curve. In all cases, the carbon allotropes improved the mechanical and thermal attributes of the polymer matrix. These studies confirm that the MD method is a practical and sufficiently accurate tool for determining the mechanical properties of nanocomposites; accordingly, it was employed in the current study.

CA contains a high density of hydroxyl groups, which confer hydrophilicity, good plasticity, a large surface area, and high compatibility with additives, thereby emphasizing its applications in gas separation and water purification^31^. However, CA suffers from relatively low mechanical stability. In contrast, polyacrylonitrile PAN is a well-known thermoplastic polymer with stable mechanical and chemical properties^32^, which can effectively compensate for the mechanical weakness of CA when incorporated into CA/PAN composites. The resulting composite inherits the advantageous properties of both CA and PAN and can be used as a promising membrane material^33^. At the interface between PAN and CA, interfacial energy plays a critical role in determining the strength and stability of surface bonding. This energy represents the work required to separate the interfacial region between the two polymers and directly influences the mechanical integrity and structural cohesion of the membrane. A lower interfacial energy indicates stronger molecular-scale interactions, thereby improving adhesion and compatibility between the braided support layer and the polymeric separation layer, which is essential for high-strength membrane applications.

This study aims to address this research gap by conducting a molecular dynamics analysis of BRHFMs designed for water treatment applications. The primary objectives are to examine the interfacial energy and bonding mechanisms between the polymer coating and the braid reinforcement, evaluate the impact of polymer homogeneity (CA/CA, PAN/PAN) and heterogeneity (CA/PAN) on mechanical performance, and provide molecular-level insights into how interfacial characteristics influence membrane strength. The results are expected to contribute to the rational design of next-generation BRHFMs with enhanced mechanical stability, interfacial compatibility, and filtration efficiency.

## Simulation method

All simulations in this study were performed using MD, a robust and precise computational technique for exploring the physical, mechanical, chemical, and interfacial behavior of materials at the atomic scale^34–36^. MD enables detailed analysis of structural evolution, stress distribution, and coupling at the nanoelectromechanical level^37^, as well as transport phenomena such as particle diffusion^38^, providing a microscopic understanding of system behavior under various conditions. In this work, the MD framework was used to examine three key aspects: mechanical performance, interfacial interactions, and water purification mechanisms. Each of these required specific modeling strategies, simulation parameters, and analytical procedures, which are detailed in the following subsections.

The initial molecular configurations were constructed using a combination of custom-developed scripts, Packmol^39^ for molecular spatial arrangement, and Visual Molecular Dynamics (VMD)^40^ for preliminary visualization and geometry optimization. All dynamic simulations were executed using the Large-scale Atomic/Molecular Massively Parallel Simulator (LAMMPS)^41^, while structural analyses and visualizations were carried out with the Open Visualization Tool (OVITO)^42^. Post-processing and quantitative analyses were performed using in-house scripts in conjunction with OVITO to extract the desired structural and mechanical parameters from the simulation trajectories. More information on these aspects can be found elsewhere^43^.

### Young’s modulus of the braid layer

**Fig. 1:**
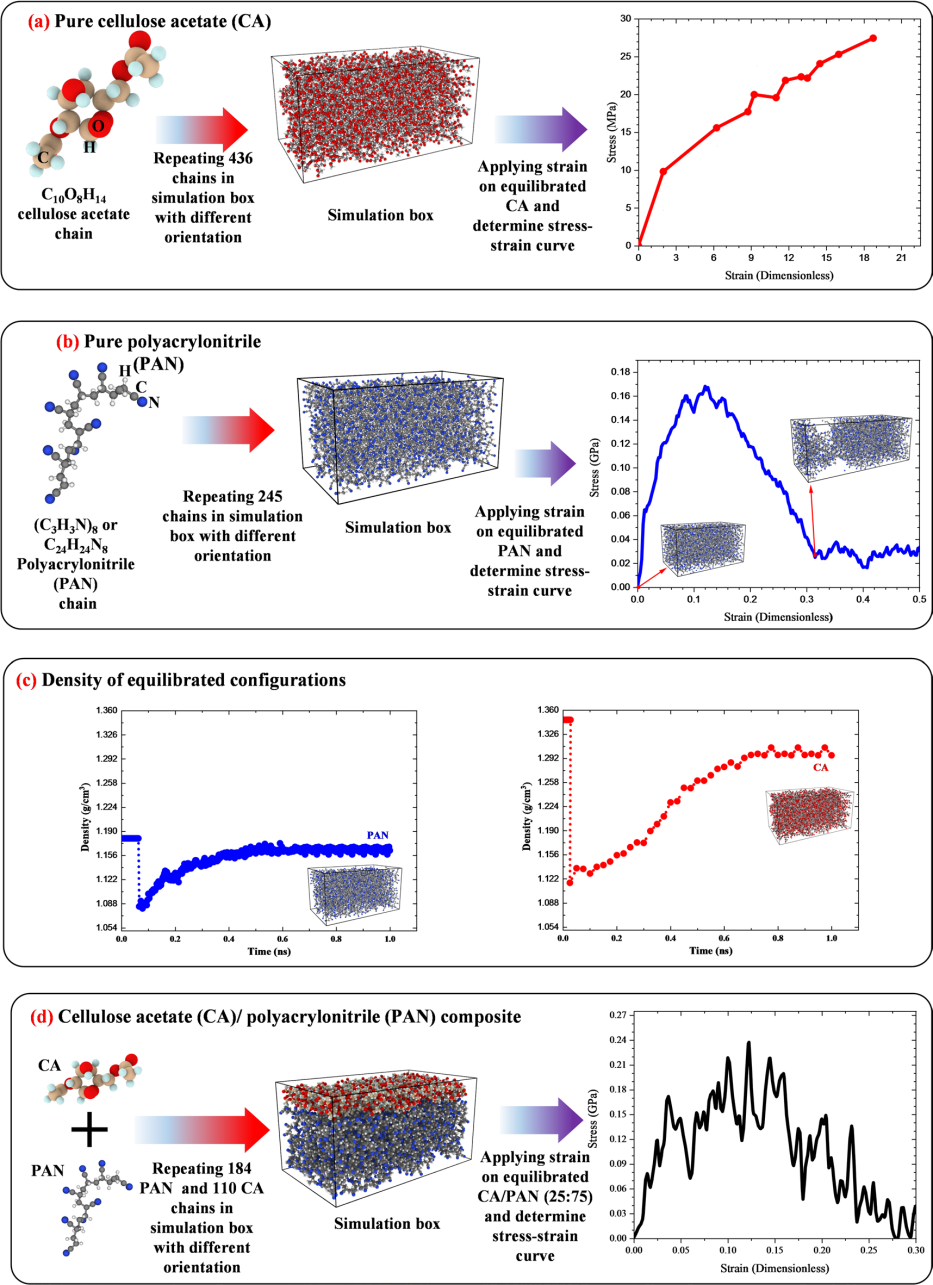
Initial configuration and stress-strain curve of (**a**) pure CA, (**b**) pure PAN, and (**d**) CA/PAN (25:75) composites. (**c**) Density of equilibrated CA and PAN.

In the MD simulation, due to the braid’s large dimensions, a polymer layer of the same density is generated to measure the mechanical properties of the braid layers. In BR-HFM systems, the mechanical strength of the membranes depends on the braid structure; therefore, the mechanical analysis focuses solely on the braid layers. To examine the mechanical behavior of CA, PAN, and their composite (CA/PAN), each system was individually constructed and simulated under identical conditions. The molecular structures of the initial polymer chains are presented schematically in Fig. 1. For the pure systems, three-dimensional simulation boxes were generated with dimensions of 4.5 × 8.0 × 4.0 nm^3^ for CA and 4.75 × 8.20 × 4.32 nm^3^ for PAN.

The CA region was filled with 436 CA chains, corresponding to a density of 1.32 g/cm^3^, while the PAN region was filled with 245 PAN chains, corresponding to a density of 1.18 g/cm^3^. These densities were taken from previous experimental studies^44,45^ to define a configuration as closely as possible to reality. In addition, to provide a benchmark for the density values, the densities of CA and PAN were computed separately using MD simulations by dividing the mass of the simulation box by its volume in individual simulations conducted under the isothermal–isobaric (NPT) ensemble at 298 K and atmospheric pressure. The densities obtained from the MD simulations were close to the experimental values, thereby confirming their reliability.

For the CA/PAN composite, an initial weight ratio of 25:75 (CA: PAN) was adopted, with the PAN weight fraction 3 times that of CA. This configuration helps to model a thick PAN layer adjacent to a thin CA layer, thereby mimicking the experimental setup^44,46^. Unfortunately, realistic configurations of BRHFMs at the micrometer scale are not feasible in molecular dynamics simulations due to impractical computational costs. As an alternative, simplified thick/thin layered configurations can be employed as representative candidates for BRHFMs.

The composite system was constructed in a simulation domain measuring 4.8 × 8.2 × 4.2 nm^3^, where 184 PAN chains were arranged at the lower region of the box, and 110 CA chains were placed above them, forming a vertically stacked bilayer configuration as depicted in Fig. 1. Periodic boundary conditions were imposed in all Cartesian directions throughout the entire simulation to reproduce bulk polymer behavior and eliminate surface effects. Interatomic interactions for all systems, including pure polymers and their composites, were described using the second-generation Reactive Force Field (ReaxFF)^46,47^, whose parameters were developed by van Duin et al. in 2010^48^. This potential function provides an accurate representation of chemical bonding and bond-breaking phenomena, effectively bridging quantum-mechanical insights with classical molecular mechanics to capture both structural and reactive behaviors within polymeric materials^34,46,47^. With respect to these features, this potential was employed; however, it is slower than other classical potentials when applied to large systems. The present study focuses on mechanical and interfacial characteristics; therefore, bond formation and breakage phenomena are not discussed in detail.

The simulation workflow, corresponding to the configurations illustrated in Fig. 1, comprised three sequential stages: energy minimization, curing–equilibration, and tensile deformation. In the energy minimization stage, a time step of 0.1 fs was employed^49^. The total potential energy of each system was minimized in the microcanonical (NVE) ensemble, with a Langevin thermostat at 1 K, for 1 ps, ensuring a relaxed, energetically stable configuration. The time step was then increased to 0.25 fs, which is sufficiently small to capture the formation and breakage of chemical bonds accurately^34^. Curing refers to the cross-linking process in a polymer, achieved by increasing the polymer’s temperature to near its melting point, maintaining it at this temperature for a sufficient period, and then cooling to room temperature. In the heating section, the temperature was gradually increased from 1 K to 500 K over 2.5 ps to activate molecular rearrangements and relieve internal stresses. The system was then maintained at 500 K and 1 bar for 1 ns to allow complete curing.

In the cooling part, the temperature was decreased to ambient conditions (~ 298 K) over 2.5 ps and held for an additional 1 ns to achieve complete thermodynamic equilibration. This rate provides a cost-effective simulation time while successfully reproducing conditions that yield experimental densities. In addition, both lower and higher cooling rates were tested; however, the selected rate (80.5 K.ps^− 1^) achieves good accuracy while significantly reducing computational cost. At the end of this stage, the simulated densities of pure CA and PAN were consistent with reported experimental values, confirming the structural stability of the equilibrated configurations and their readiness for mechanical testing. This cycle is referred to as the curing–equilibration stage under the NPT ensemble at atmospheric pressure, with a time step of 0.25 fs. Figure 1. (c) shows the densities of CA and PAN as functions of time over a 1 ns period and demonstrates that the systems reach thermodynamic equilibrium within 1 ns, indicating that longer simulation times are not required.

During the tensile deformation stage, uniaxial tension was applied along the longitudinal axis of the simulation box at a constant strain rate of 10^− 3^ ps^− 1^^23,50,51^. The simulation box itself was elongated in the same direction at the same rate to preserve homogeneous deformation and prevent artificial void formation. To avoid ensemble-induced artifacts, the tensile direction was decoupled from the NPT ensemble during loading. The virial stress was computed along the tensile axis, incorporating both kinetic and potential (virial) components. The resulting stress–strain data were used to construct the corresponding stress–strain curves, from which Young’s modulus (Y) and fracture characteristics were derived. More information regarding this method can be found elsewhere^23,50,52–54^.

Figures 1.a, 1.b, and 1.d show the stress–strain curves of pure CA, pure PAN, and the CA/PAN (25:75) composite, respectively. These curves were used to extract Young’s modulus from the elastic region of the diagrams. Because the tensile response of materials depends on their nature, the elastic region differs for each material. Accordingly, the elastic regions for CA, PAN, and the CA/PAN (25:75) composite were determined to be 6%, 0.7%, and 1%, respectively. The slopes of the stress–strain curves within these elastic regions were then calculated to determine Young’s modulus. Burst strength is also calculated at the point at which the system yields, commonly known as the ultimate strength. The stress at the yielding point represents the burst strength. The same computational algorithm was applied to all three systems (pure CA, pure PAN, and the CA/PAN composite), ensuring comparable initial equilibration and consistent mechanical loading conditions. This unified approach enabled direct, reliable evaluation of the elastic and failure behavior of each system.

### Simulation setups for interfacial characteristics

In systems composed of PAN and CA, the interfacial energy is primarily governed by intermolecular forces, including hydrogen bonding, van der Waals interactions, and dipole–dipole interactions. Functional groups such as hydroxyl and acetate in CA and nitrile groups in PAN play a key role in forming strong interfacial interactions and enhancing phase miscibility. However, quantitative information on the exact interfacial energy between PAN and CA remains limited. To address this, MD simulations were conducted to evaluate two primary aspects: (i) the cohesive or interfacial energy between the polymeric phases, and (ii) the mechanical stability of the resulting interface. The preliminary steps of energy minimization and curing–equilibration followed the methodology described in Sect.  2.1 to ensure structural relaxation and thermal equilibrium before interfacial analysis.

**Fig. 2:**
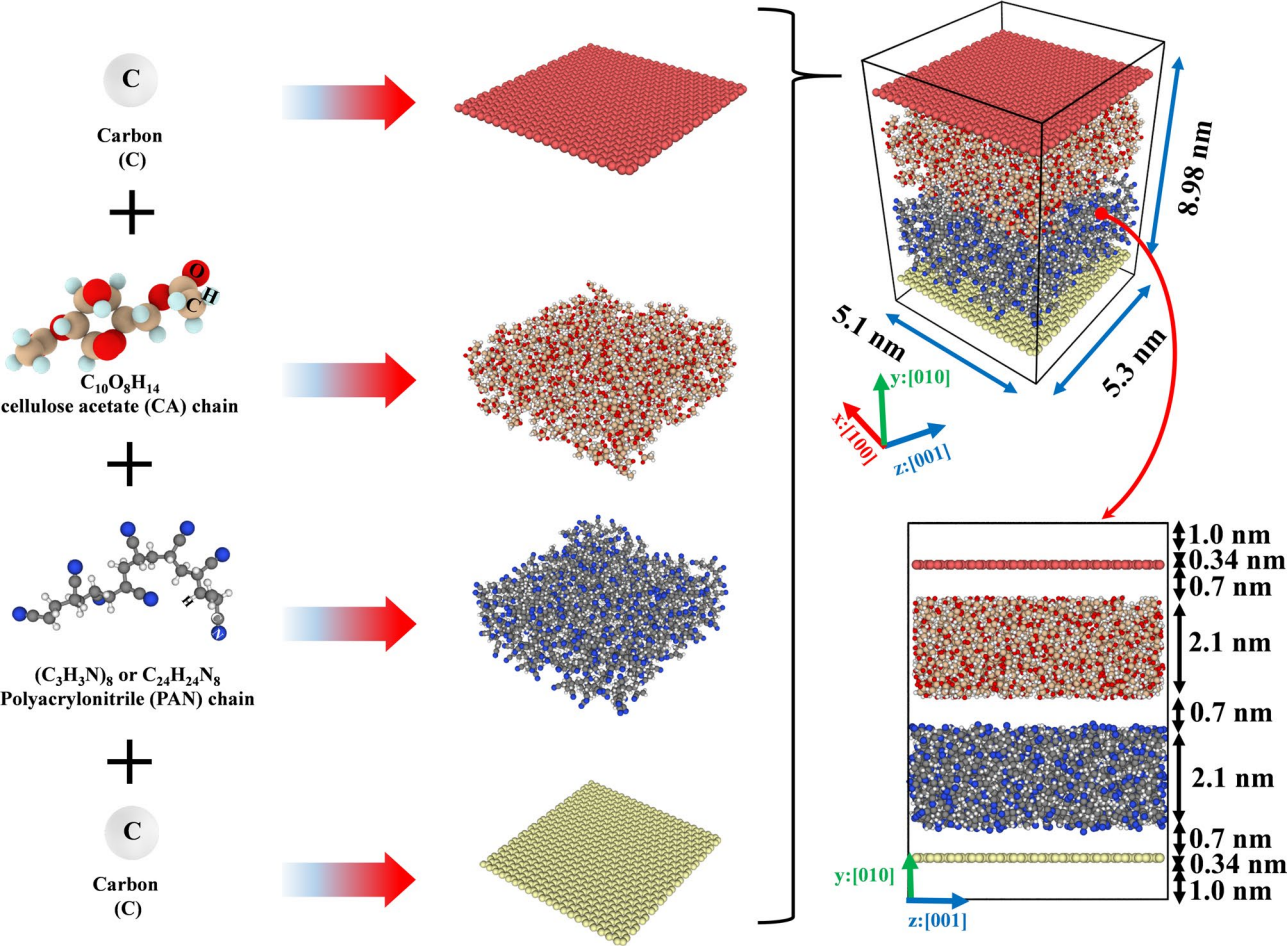
Process of constructing the CA/PAN composite and its initial structure.

A CA/PAN composite with an equal weight ratio (50:50) was constructed as the interfacial model. The configuration was arranged such that the PAN phase occupied the lower half of the box and the CA phase the upper half, forming a distinct interfacial boundary between the two polymers. To control compression and facilitate interface formation, the entire system was confined between two graphene pistons, as shown in Fig. 2. The pistons were driven toward each other under an applied pressure of 1 bar at 500 K, maintained via a Nosé–Hoover thermostat in the canonical (NVT) ensemble. To compress the CA and PAN and push them toward each other, the NVT ensemble was applied to the polymers rather than to the graphene piston. In LAMMPS, the “aveforce” command was used to move the piston according to a pressure of 1 bar. In this method, the force applied to each carbon atom in the graphene piston was calculated by multiplying the graphene surface area by the applied pressure and dividing by the total number of carbon atoms. This force was applied to all carbon atoms in the graphene, causing the piston to move along the direction of the applied force. In this approach, no thermostat or barostat was applied to the graphene; the NVT thermostat was applied only to the polymer region. This compression brought the CA and PAN chains into close contact, enabling molecular interdiffusion and interfacial bonding, thereby promoting the natural development of an adhesive interface.

**Fig. 3:**
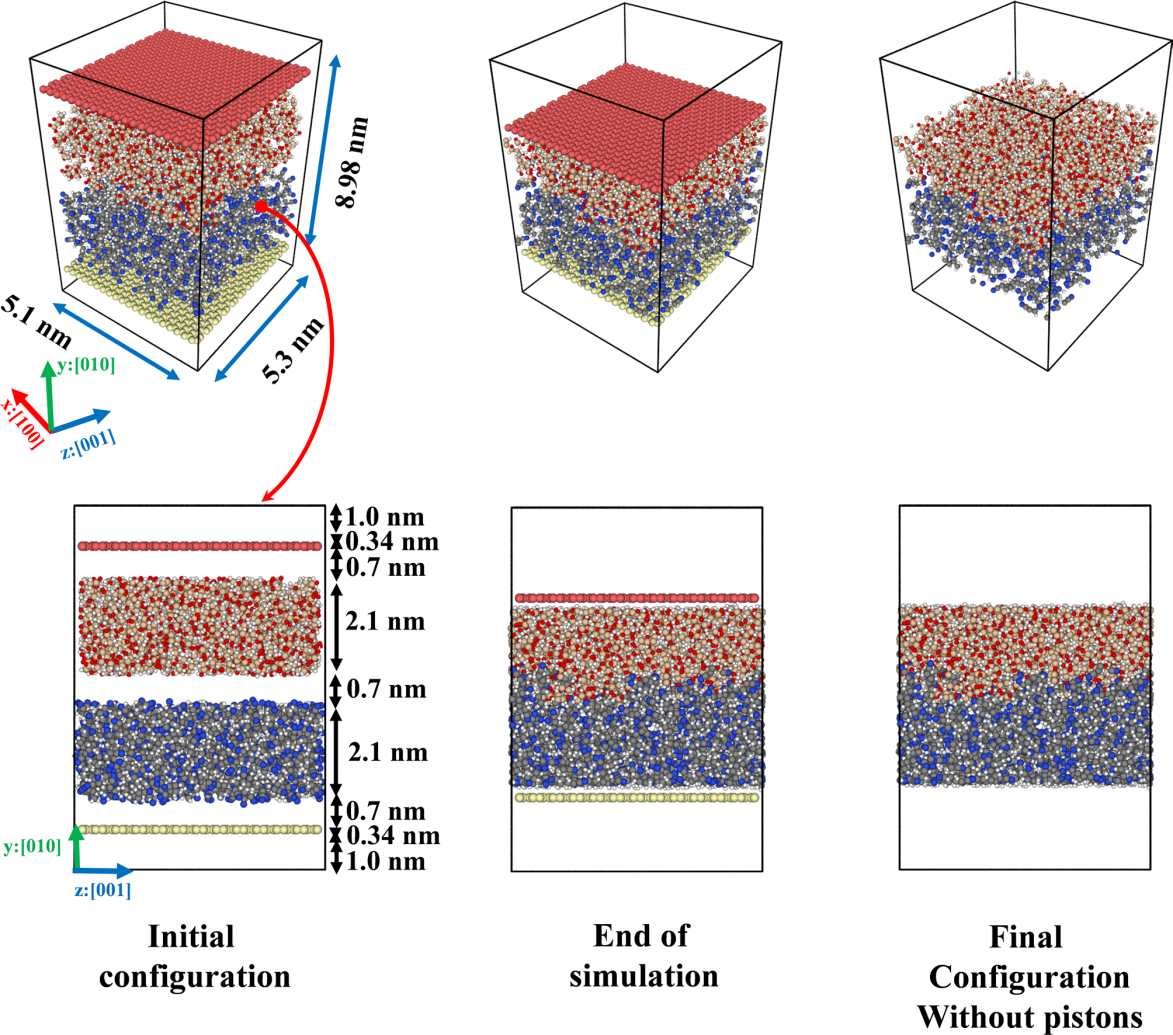
The snapshot of the CA/PAN configuration at the initial and final steps of the simulation, as well as after removing the graphene pistons. The final configuration without a piston was used to calculate interfacial energy and mechanical stability.

After the formation of chemical bonds and intimate contact between the polymer phases, the system underwent a curing–equilibration stage identical in duration and temperature profile to that of the mechanical simulations, allowing complete relaxation of interfacial stresses. Once equilibrium was reached, the graphene pistons were carefully removed, yielding a self-standing CA/PAN composite with a well-defined and relaxed interfacial region, suitable for subsequent evaluation of interfacial strength and energy. The final equilibrated configuration of the composite system is illustrated in Fig. 3. Regarding the reproducibility and reliability of the model used to create linkages between CA and PAN, it is worth noting that the algorithm was executed 5 times with different random-velocity seed values to evaluate its accuracy. More information regarding this method can be found elsewhere^52,55,56^. In each case, the simulations were performed independently with distinct velocity seeds, and the interfacial energy was subsequently computed. Across all cases, the calculated values were in close agreement, with a deviation of approximately ± 0.02 eV.

After obtaining the equilibrated CA/PAN composite, the interfacial energy was calculated following the procedure proposed by Jiang et al.^57^ and other researchers^58,59^. In this method, the total potential energy of the composite system ($\:{\mathrm{E}}_{\mathrm{S}\mathrm{y}\mathrm{s}\mathrm{t}\mathrm{e}\mathrm{m}}$) was first evaluated, as illustrated in Fig. 3. The energy of each component will then be reduced accordingly, as described in Eq. (1).

1 $$\:{\mathrm{E}}_{\mathrm{I}\mathrm{n}\mathrm{t}\mathrm{e}\mathrm{r}}={\mathrm{E}}_{\mathrm{S}\mathrm{y}\mathrm{s}\mathrm{t}\mathrm{e}\mathrm{m}}-({\mathrm{E}}_{\mathrm{P}\mathrm{A}\mathrm{N}}+{\mathrm{E}}_{\mathrm{C}\mathrm{A}})$$

Here, $\:{\mathrm{E}}_{\mathrm{i}\mathrm{n}\mathrm{t}\mathrm{e}\mathrm{r}}$ is the interfacial energy between CA and PAN; the energies of PAN and CA have been denoted by $\:{\mathrm{E}}_{\mathrm{P}\mathrm{A}\mathrm{N}}$ and $\:{\mathrm{E}}_{\mathrm{C}\mathrm{A}}$, respectively. There are two methods to compute $\:{\mathrm{E}}_{\mathrm{P}\mathrm{A}\mathrm{N}}$ and $\:{\mathrm{E}}_{\mathrm{C}\mathrm{A}}$. In the first method^57^, these energies are computed using two separate simulations: one for a pure CA system and one for a pure PAN system, each with the same mass and volume as the corresponding phase in the composite. The potential energies of these individual systems are then used in Eq. 1. In the second method^58,59^, the energies $\:{\mathrm{E}}_{\mathrm{C}\mathrm{A}}$ and $\:{\mathrm{E}}_{\mathrm{C}\mathrm{A}}$ are computed directly from the original molecular configuration by grouping atoms within a single MD simulation, simultaneously with $\:{\mathrm{E}}_{\mathrm{S}\mathrm{y}\mathrm{s}\mathrm{t}\mathrm{e}\mathrm{m}}$. The second MD-based approach can yield more accurate results. In contrast, the first mimics the methodology commonly used in quantum-mechanical simulations^61,62^. In this regard, the first approach was employed in the present study to determine the interfacial energy. In this work, it is noted that the energies used as inputs in Eqs. (1)–(3) were computed over a 100 ps interval, and their average values were employed. This approach uses energy values that are independent of microstate fluctuations the system may undergo at specific times.

A negative value of $\:{\mathrm{E}}_{\mathrm{i}\mathrm{n}\mathrm{t}\mathrm{e}\mathrm{r}}$​ indicates a thermodynamically favorable interface, implying good adhesion between the two polymer phases. For comparison, a homogeneous CA–CA system and a hybrid configuration combining a CA layer with a CA–PAN braid were analyzed. Their interfacial energies were computed as: Their interfacial energies were computed as:

2 $$\:{\mathrm{E}}_{\mathrm{I}\mathrm{n}\mathrm{t}\mathrm{e}\mathrm{r}}={\mathrm{E}}_{\mathrm{S}\mathrm{y}\mathrm{s}\mathrm{t}\mathrm{e}\mathrm{m}}-\left(2{\mathrm{E}}_{\mathrm{C}\mathrm{A}}\right)$$

3 $$\:{\mathrm{E}}_{\mathrm{I}\mathrm{n}\mathrm{t}\mathrm{e}\mathrm{r}}={\mathrm{E}}_{\mathrm{S}\mathrm{y}\mathrm{s}\mathrm{t}\mathrm{e}\mathrm{m}}-({\mathrm{E}}_{\mathrm{P}\mathrm{A}\mathrm{N}|\mathrm{C}\mathrm{A}}+{\mathrm{E}}_{\mathrm{C}\mathrm{A}})$$

**Fig. 4:**
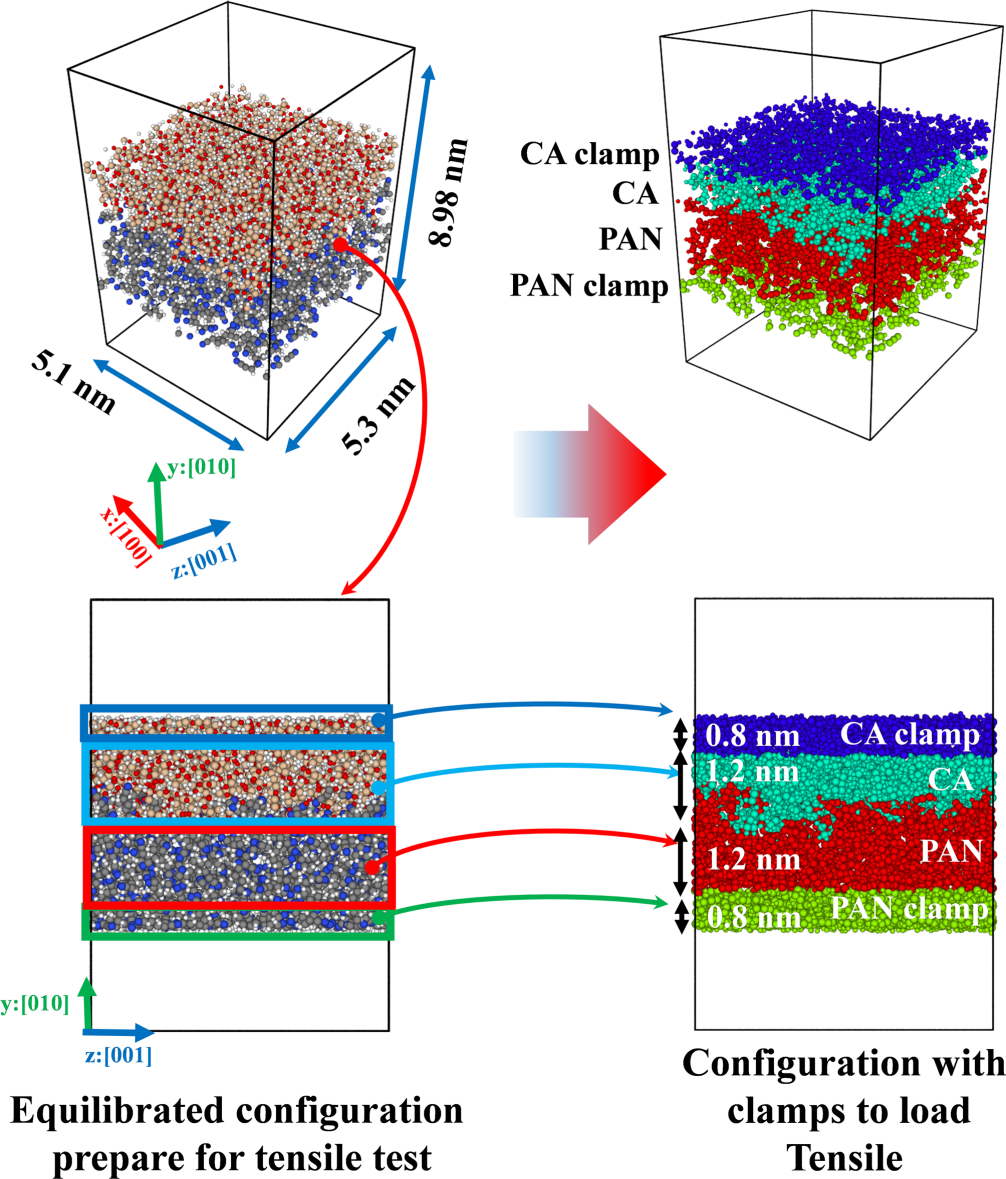
The CA/PAN configuration with a clamped-clamped geometry to apply a tensile load perpendicular to the CA/PAN composite interface.

In this study, three interfacial configurations were examined: one heterogeneous (CA/PAN), two homogeneous (CA–CA and PAN–PAN), and one hybrid structure (CA layer combined with a CA–PAN braid). This comparison allowed for a clear assessment of how interfacial composition influences the cohesion, adhesion strength, and mechanical integrity of the composite membranes. To further evaluate the mechanical stability of the interphase, a uniaxial tensile load was applied perpendicular to the interface of the equilibrated CA/PAN composite. As shown in Fig. 4, two boundary regions were designated as clamps, one in the upper CA domain and the other in the lower PAN domain. Both clamps were rigidly constrained, and a constant velocity of 10^− 3^ ps^− 1^ was imposed in opposite directions, causing them to move apart and generate tensile stress normal to the interfacial plane.

Throughout the loading process, the temperature of the remaining system (excluding the fixed clamps) was regulated using the canonical (NVT) ensemble at ambient temperature solely for thermal control. This temperature regulation aimed to dissipate excess heat produced during deformation and does not indicate thermodynamic equilibrium, as the tensile test is inherently a non-equilibrium molecular dynamics simulation. The stress evolution during deformation was calculated using the same virial formulation described earlier, allowing the generation of the corresponding stress–strain response. This setup facilitated a detailed analysis of interfacial deformation and separation mechanisms, thereby revealing the intrinsic adhesive strength and failure behavior of the CA/PAN interface under tensile stress.

## Results and discussion

To evaluate the mechanical integrity of the membrane materials, the tensile and burst strengths of three representative structures (pure CA, pure PAN, and the hybrid CA/PAN composite) were analyzed through MD simulations. The corresponding results are summarized in Table 1. The Young’s modulus values obtained for these systems were 0.231 GPa for CA, 3.0 GPa for PAN, and 2.73 GPa for the CA/PAN composite. These results are in good agreement with both the experimental data reported in the literature^45,63–66^ and the theoretical predictions derived from the Karkkainen et al. composite modulus model (Eqs. (4)–(7))^23,67^. In these equations, $\:{\mathrm{Y}}_{\mathrm{C}\mathrm{o}\mathrm{m}\mathrm{p}\mathrm{o}\mathrm{s}\mathrm{i}\mathrm{t}\mathrm{e}}$​ represents the overall Young’s modulus of the composite, $\:{\mathrm{Y}}_{\mathrm{P}\mathrm{A}\mathrm{N}}$​ and $\:{\mathrm{Y}}_{\mathrm{C}\mathrm{A}}$​ are the Young’s moduli of the PAN and CA components, respectively, and $\:{\mathrm{f}}_{\mathrm{P}\mathrm{A}\mathrm{N}}$​ and $\:{\mathrm{f}}_{\mathrm{C}\mathrm{A}}$​ denote their corresponding volume fractions within the composite. The sum of the volume fractions equals one, indicating that the composite is entirely composed of these two constituents.

4 $$\:{\mathrm{Y}}_{\mathrm{C}\mathrm{o}\mathrm{m}\mathrm{p}\mathrm{o}\mathrm{s}\mathrm{i}\mathrm{t}\mathrm{e}}={\mathrm{f}}_{\mathrm{P}\mathrm{A}\mathrm{N}}{\mathrm{Y}}_{\mathrm{P}\mathrm{A}\mathrm{N}}+{\mathrm{f}}_{\mathrm{C}\mathrm{A}}{\mathrm{Y}}_{\mathrm{C}\mathrm{A}}$$

5 $$\:{\mathrm{f}}_{\mathrm{P}\mathrm{A}\mathrm{N}}=\frac{{\mathrm{V}}_{\mathrm{P}\mathrm{A}\mathrm{N}}}{{\mathrm{V}}_{\mathrm{C}\mathrm{o}\mathrm{m}\mathrm{p}\mathrm{o}\mathrm{s}\mathrm{i}\mathrm{t}\mathrm{e}}}$$

6 $$\:{\mathrm{f}}_{\mathrm{C}\mathrm{A}}=\frac{{\mathrm{V}}_{\mathrm{C}\mathrm{A}}}{{\mathrm{V}}_{\mathrm{C}\mathrm{o}\mathrm{m}\mathrm{p}\mathrm{o}\mathrm{s}\mathrm{i}\mathrm{t}\mathrm{e}}}$$

7 $$\:{\mathrm{f}}_{\mathrm{P}\mathrm{A}\mathrm{N}}+{\mathrm{f}}_{\mathrm{C}\mathrm{A}}=1$$

Using $\:{\mathrm{f}}_{\mathrm{P}\mathrm{A}\mathrm{N}}$​=0.75, $\:{\mathrm{f}}_{\mathrm{C}\mathrm{A}}$​=0.25, $\:{\mathrm{Y}}_{\mathrm{P}\mathrm{A}\mathrm{N}}$​=3.0 GPa, and $\:{\mathrm{Y}}_{\mathrm{C}\mathrm{A}}$​=0.231 GPa, the theoretical modulus of the CA/PAN composite is calculated as $\:{\mathrm{Y}}_{\mathrm{C}\mathrm{o}\mathrm{m}\mathrm{p}\mathrm{o}\mathrm{s}\mathrm{i}\mathrm{t}\mathrm{e}}$​=2.30 GPa, which is in close agreement with the MD-predicted value of 2.73 GPa. This good consistency confirms the validity and accuracy of the MD simulation methodology and the reliability of the employed force field (ReaxFF) in reproducing realistic mechanical responses. Table 1 compares the Young’s modulus and burst strength of pure CA and PAN in their bulk phases with values reported in the literature, as well as those of the CA/PAN (25:75) composite with reported experimental data. For pure CA and PAN, the properties derived from molecular dynamics simulations and those reported in the literature correspond to bulk phases and are therefore comparable. Moreover, for the composite, because the weight ratio used in the current simulations matches that in the experimental studies, the results are directly comparable.

The analysis of burst (tensile rupture) strength, as summarized in Table 2, also supports the reinforcement effect of the PAN braid. The pure CA layer exhibited a burst strength of 0.83 MPa, reflecting its high flexibility but low stiffness. In contrast, the pure PAN layer showed a higher stiffness (3 GPa modulus) but a lower burst strength of 0.24 MPa, indicating its brittle nature under multiaxial stress. The hybrid structure containing 75% PAN / 25% CA achieved an intermediate burst strength of 0.40 MPa, representing an optimal balance between tensile rigidity and flexibility. The weight fractions of the braided layers in CA/CA (50:50), PAN/PAN (50:50), and CA/PAN (25:75) composites show that increasing the CA weight fraction results in higher burst strength. Conversely, reducing the CA content and increasing the PAN fraction decreases the burst strength.

**Table 1:** Comparison of the Young’s modulus obtained from MD simulation with theoretical and experimental data for CA, PAN, and CA/PAN composite structures. The Young’s modulus listed in this table was determined from the stress–strain curve illustrated in Fig. 1.

No.	System	Young’s Modulus	Density (g/cm^3^)	Method/Source Description	Reference
1	CA bulk	231 MPa	1.29 ± 0.03	MD simulation	This study
2		250 MPa	1.32^a^	Experiment	^68^
3		248 MPa	1.32 ^a^	Experiment	^64^
4		375 MPa	1.32 ^a^	Experiment	^65^
5		205 MPa	1.32 ^a^	Braided CA, Experiment, (Fan et al.)	^45^
1	PAN bulk	3 GPa	1.16 ± 0.02	MD simulation	This study
2		2–3 GPa	1.18-1.20^a^	Experiment	^66,67^
3		2.65 GPa	1.18-1.20^a^	Braided PAN, Experiment, (Fan et al.)	^44^
1	CA/PAN (25:75) composite	2.73 GPa	-	MD simulation	This study
2		2.30 GPa	-	Karkkainen et al. Model, Theory	^23,69^
3		2.20 GPa	-	Braided CA/PAN, Experiment, (Fan et al.)	^44^

a: Ref^44,45^.

**Table 2:** Comparison of the burst (tensile failure) strength of CA, PAN, and CA/PAN braid layers obtained from MD simulation and experimental results.

No.	Braided Layer	Weight ratio	Burst Strength (MPa)	Reference
1	(CA/CA)	(50:50)	0.83	This study
2	(CA/CA) (Fan et al.)	(50:50)	0.76	^45^
3	(PAN/PAN)	(50:50)	0.241	This study
4	(PAN/PAN) (Fan et al.)	(50:50)	0.22	^45^
5	(CA/PAN)	(25/75)	0.399	This study
6	(CA/PAN) (Fan et al.)	(25/75)	0.34	^45^

**Table 3:** Comparison of stress drop points of CA, PAN, and CA/PAN braid layers obtained from MD simulation and experimental results. The stress drop points were determined from Fig. 7.

No.	Braided Layer	Weight ratio	Stress drop points (MPa)			Reference
			First	Secound	Third	
1	(CA/CA)	(50:50)	27	41	48	This study
6	(CA/PAN) (Fan et al.)	(25/75)	128	164	185	This study

These findings demonstrate that increasing the PAN content within the braid structure significantly enhances the overall tensile strength of the BR-HFM, while maintaining adequate ductility. The hybrid configuration thus offers a promising design for mechanically strong, durable membranes capable of withstanding high mechanical loads in industrial filtration and water treatment applications. Table 3. compares the stress drop points of the CA/CA and CA/PAN braid layers extracted from Fig. 7 for the first three drops. Comparison of these drop points reveals that the CA/PAN braid layer exhibits higher stress drop points than the CA/CA braid layer due to the presence of PAN, which enhances the mechanical strength of the braid layer.

**Fig. 5:**
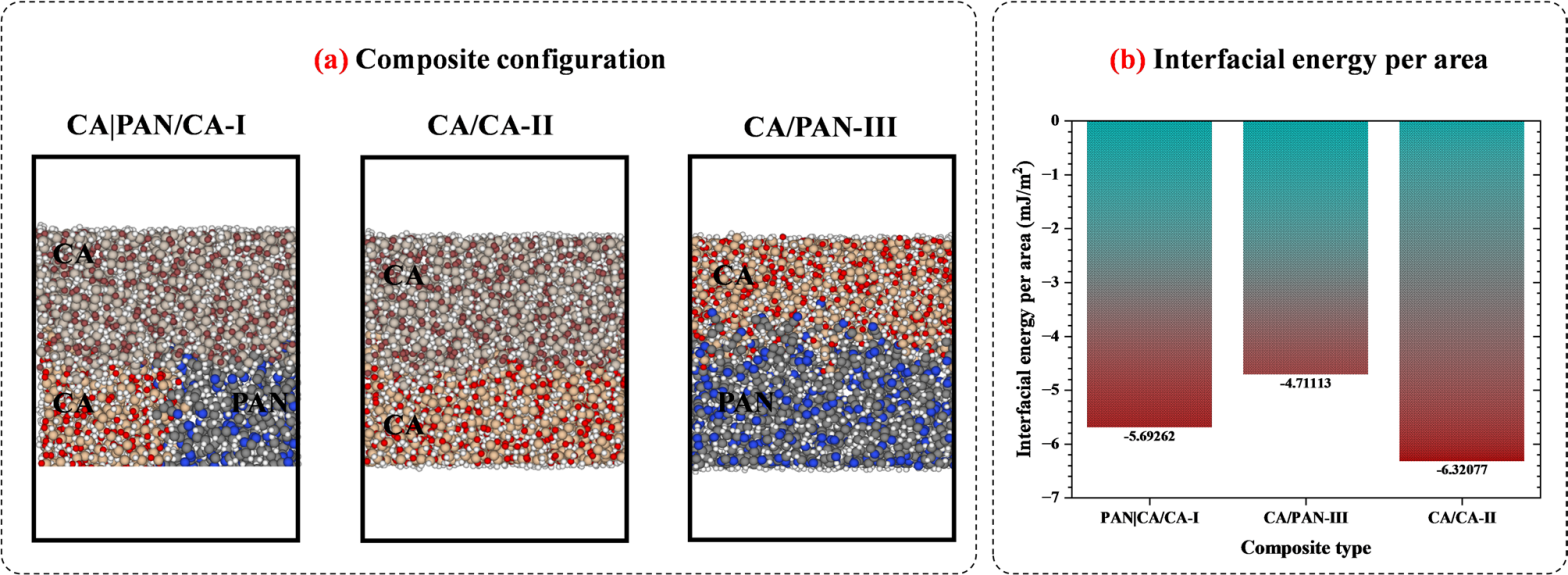
(**a**) Initial configuration and (**b**) interfacial energy of various composite structures (CA/PAN–III, PAN|CA/CA–I, and CA/CA–II) from molecular dynamics simulations.

Figure 5 presents the calculated interfacial energies per unit surface area for three composite configurations: PAN|CA/CA–I, CA/CA–II, and CA/PAN–III. The corresponding interfacial energies per area were − 5.69262, − 6.32077, and − 4.71113 mJ/m^2^, respectively. As shown in the figure, the CA/CA–II configuration demonstrates the strongest interfacial cohesion, followed by PAN|CA/CA–I, while CA/PAN–III exhibits the weakest interfacial adhesion and structural stability. A lower interfacial energy (i.e., more negative) indicates stronger bonding and greater stability between the polymer layers. The high stability of the CA/CA–II structure can be attributed to the chemical homogeneity of the two CA layers, which consist of identical polymer chains bearing hydroxyl (–OH) and acetate (–OCOCH_3_) groups. This molecular similarity promotes strong hydrogen bonding and van der Waals interactions at the interface, thereby enhancing adhesion and reducing interdiffusion between the layers.

In contrast, the PAN|CA/CA–I system shows lower interfacial stability than CA/CA–II, mainly because of the chemical difference between the two polymer phases. The nitrile (–CN) groups in PAN produce dipolar interactions that are naturally weaker than the hydrogen bonds within CA, leading to decreased compatibility and weaker interfacial cohesion. The CA/PAN–III hybrid structure has the lowest interfacial energy. This reduction results from the poor compatibility between the polar –CN groups in PAN and the acetate (–OCOCH_3_) groups in CA, which causes weaker molecular attractions and reduced hydrogen-bonding ability. As a result, the interface becomes more diffuse and less cohesive, with increased molecular mobility across the boundary. Overall, Fig. 5 clearly demonstrates that interfacial adhesion strength decreases in this order: CA/CA–II > PAN|CA/CA–I > CA/PAN–III.

This trend confirms that homogeneous polymer interfaces demonstrate superior adhesion and structural stability due to molecular compatibility, while heterogeneous or hybrid systems face interfacial discontinuities and weaker bonding. In the context of BR-HFMs, this finding indicates that although hybrid layers can enhance flexibility, their interfacial bonding remains inherently weaker. Therefore, optimizing the chemical compatibility between the braid and the selective polymer layer is crucial for developing mechanically robust and durable membrane structures.

**Fig. 6:**
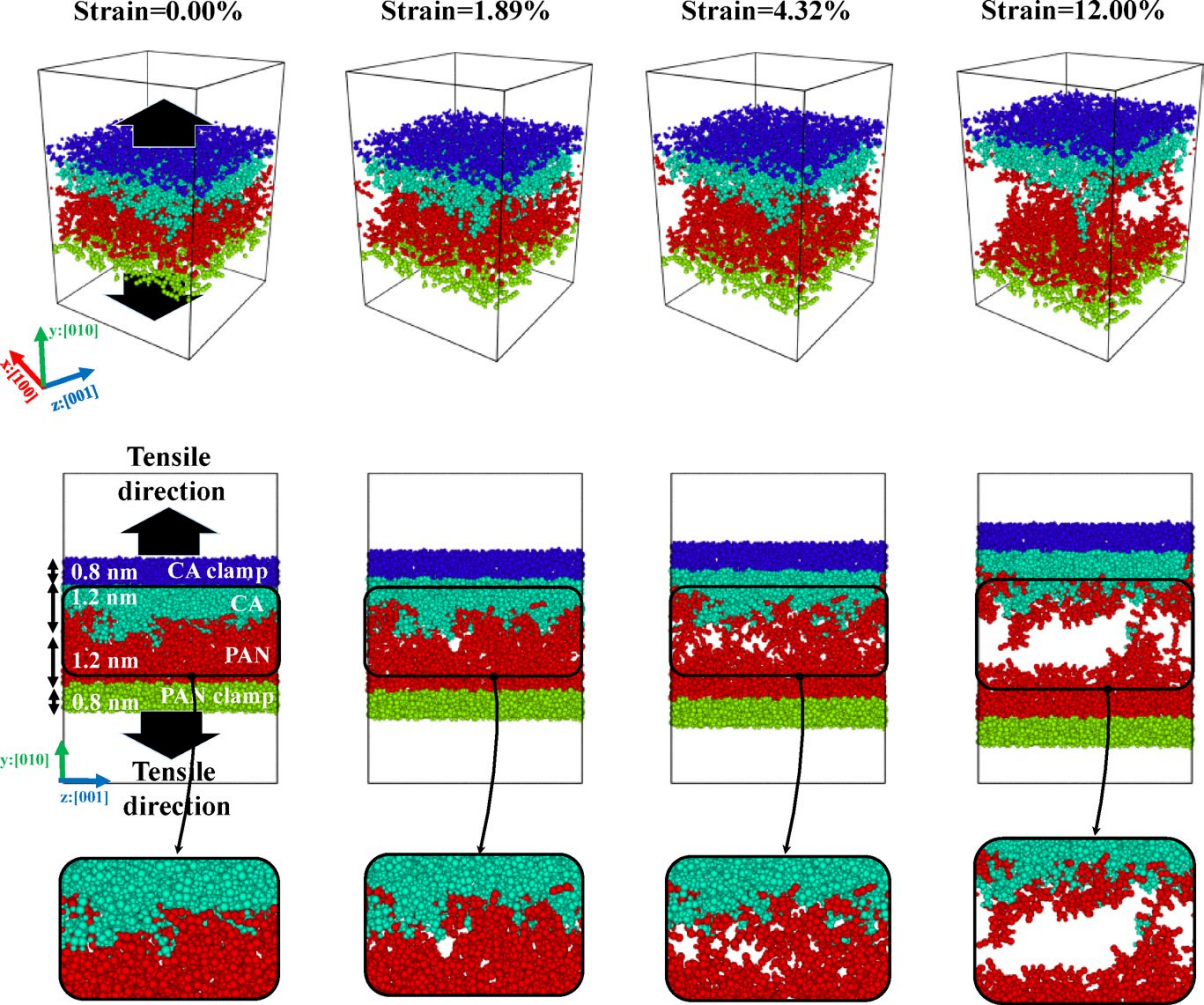
Snapshot images of the PAN/CA composite under strain at various strain percentages at 298 K.

Figure 6 shows the behavior of the heterogeneous PAN/CA-III composite from the start of the simulation (zero strain) to the point of complete yielding, which corresponds to the full separation of the two polymers at the interface at a temperature of 298 K. This figure clearly indicates that although PAN is stiffer than CA, failure occurs within the PAN region rather than at the PAN–CA interface or within the CA region. This unexpected failure pattern can be explained by differences in burst strength. As shown in Table 2, the burst strength of PAN is lower than that of CA and the CA/PAN composite, resulting in a more brittle response under multiaxial stress. As a result, failure is more likely to happen in the PAN region than in other parts of the composite. For the other composites with different weight fractions studied in this research, similar behavior was observed. In all cases, the composite yielded in the PAN region rather than in other areas.

**Fig. 7:**
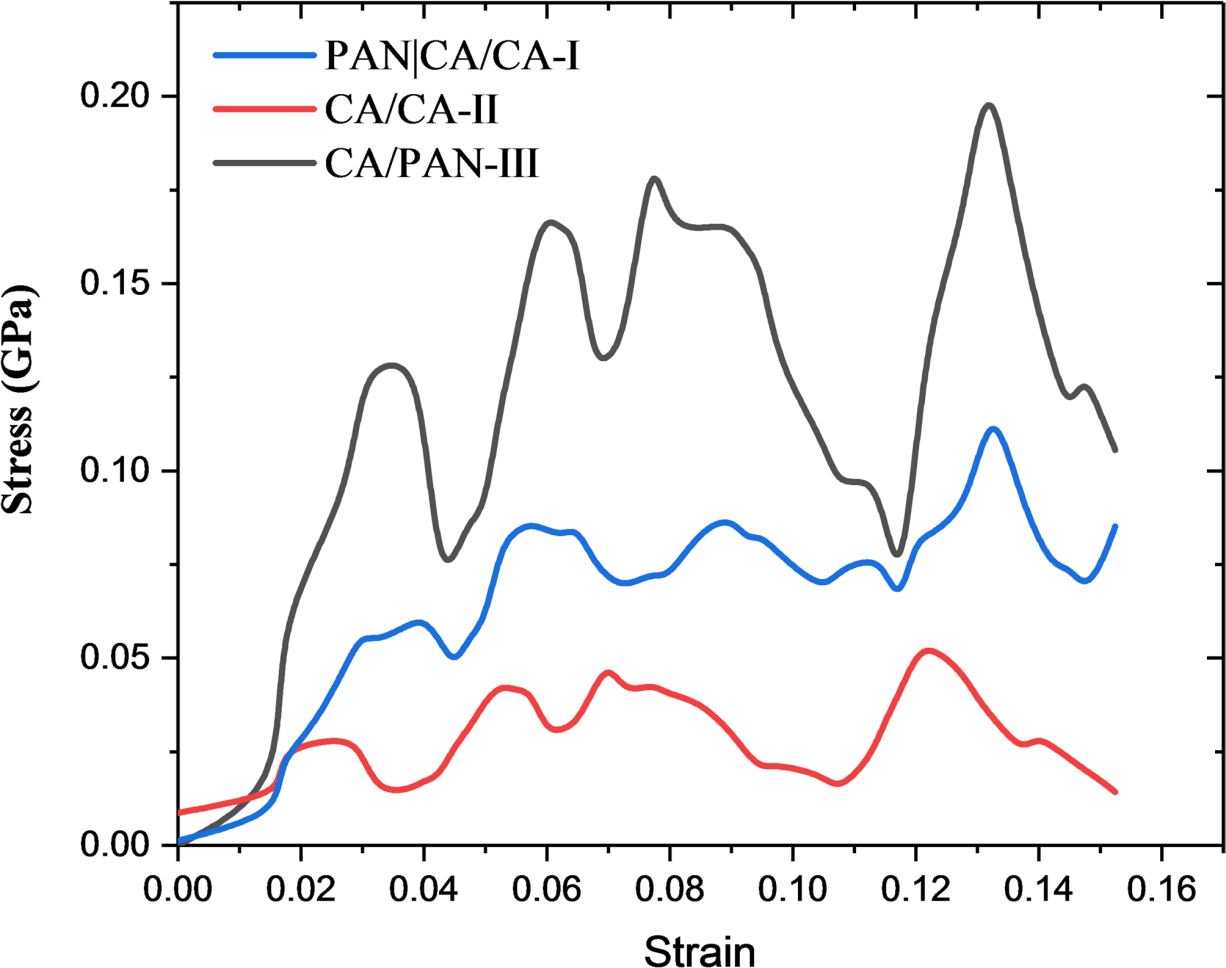
Comparison of stress–strain curves for three composites: PAN|CA/CA-I, CA/CA-II, and CA/PAN-III, under ambient temperature and pressure conditions.

Figure 7 compares the stress–strain curves of three composite structures: PAN|CA/CA-I, CA/CA-II, and CA/PAN-III, under ambient conditions. The CA/PAN-III composite shows sharp stress fluctuations, indicating strong but localized interfacial bonding that breaks suddenly under strain due to chemical and structural heterogeneity. In contrast, the PAN|CA/CA-I and CA/CA-II systems exhibit smoother stress–strain behavior, implying more gradual chain separation and weaker interfacial interactions. Overall, the heterogeneous hybrid structure (CA/PAN-III) demonstrates improved mechanical responsiveness and higher interfacial activity compared to the more homogeneous composites.

In reactive simulations performed using ReaxFF, the stress captured during tensile testing inherently includes thermal and mechanical noise arising from thermal fluctuations and bond formation/breakage events within the system. These effects introduce significant fluctuations in the stress–strain curves, making it difficult to accurately identify the main stages of yielding and fracture. Ensemble averaging during data acquisition and post-processing are two practical approaches used to reduce thermal and mechanical noise, respectively. For ensemble averaging, stress data were collected every 100 simulation steps and averaged to suppress thermal fluctuations. During post-processing, the Savitzky–Golay smoothing method [69] was applied to the stress–strain data to reduce small fluctuations associated with bond dynamics. This strategy preserves the overall trend of the stress–strain curves while removing unnecessary noise. The method was applied to the data shown in Fig. 7, yielding more precise, more interpretable diagrams.

## Conclusion

This work systematically examined the interfacial energetics and mechanical performance of CA- and PAN-based composites using molecular dynamics simulations. The results showed that interfacial adhesion strength follows the order CA/CA–II > PAN|CA/CA–I > CA/PAN–III, indicating that homogeneous polymer systems have stronger cohesive forces and better structural stability. The CA/CA–II configuration displayed the most negative interfacial energy (− 6.32077 mJ/m^2^) and the smoothest stress–strain profile, reflecting strong hydrogen bonding and van der Waals interactions. Conversely, the heterogeneous CA/PAN–III interface had a more negative energy (− 4.71113 mJ/m^2^) and noticeable stress oscillations, indicating localized strong bonding but weaker overall stability due to interfacial chemical mismatch. The Young’s modulus values for these systems were 0.231 GPa for CA, 3.0 GPa for PAN, and 2.73 GPa for the CA/PAN composite.

The mechanical simulations further demonstrated that increasing PAN content enhances the tensile modulus of BRHFMs, while CA incorporation improves flexibility. The hybrid CA/PAN structure thus achieves an effective balance between stiffness and ductility, making it an excellent candidate for membrane applications under mechanical load. Overall, this study confirms the reliability of the ReaxFF-based MD framework for predicting interfacial and mechanical properties of polymer composites and provides practical molecular-level guidance for tailoring the composition and architecture of high-performance braid-reinforced membranes.

## Data Availability

The molecular dynamics simulation data that support the findings of this study are available from the corresponding author upon reasonable request.

## Abbreviations

MD: Molecular Dynamics
BRHFMs: Braid-Reinforced Hollow Fiber Membranes
CA: Cellulose Acetate
PAN: Polyacrylonitrile
MBRs: Membrane Bioreactors
VMD: Visual Molecular Dynamics
LAMMPS: Large-scale Atomic/Molecular Massively Parallel Simulator
OVITO: Open Visualization Tool
ReaxFF: Reactive Force Field
$\:{\mathrm{E}}_{\mathrm{S}\mathrm{y}\mathrm{s}\mathrm{t}\mathrm{e}\mathrm{m}}$: Total Potential Energy of the Composite System
$$\:{\mathrm{E}}_{\mathrm{i}\mathrm{n}\mathrm{t}\mathrm{e}\mathrm{r}}$$: Interfacial Energy
